# Hepatitis C in Pregnancy

**DOI:** 10.3390/diseases6020031

**Published:** 2018-04-27

**Authors:** Pratima Dibba, Rosann Cholankeril, Andrew A. Li, Meera Patel, Mariam Fayek, Christy Dibble, Nnenna Okpara, Autumn Hines, Aijaz Ahmed

**Affiliations:** 1 Department of Gastroenterology, Women & Infants Hospital/Warren Alpert School of Medicine, Brown University, Providence, RI 02905, USA; pratima_dibba@brown.edu (P.D.); mfayek@wihri.org (M.F.); cdibble@wihri.org (C.D.); nokpara@wihri.org (N.O.); ahines@kentri.org (A.H.); 2 Department of Medicine, Roger Williams Medical Center, Providence, RI 02908, USA; rtc1088@gmail.com; 3 Division of Gastroenterology and Hepatology, Stanford University School of Medicine, Stanford, CA 94304, USA; andrewli@stanford.edu; 4 Department of Hematology/Oncology, Wake Forest Baptist Medical Center, Winston-Salem, NC 27157, USA; mrpatel@wakehealth.edu

**Keywords:** hepatitis C virus infection, pregnancy

## Abstract

The prevalence of hepatitis C in pregnancy is as high as 3.6% in large cohorts. The prevalence of hepatitis C acquired by vertical transmission is 0.2% to 0.4% in the United States and Europe. Although screening is not recommended in the absence of certain risk factors, the importance of understanding hepatitis C in pregnancy lies in its association with adverse maternal and neonatal outcomes. There is potential for those infants infected by vertical transmission to develop chronic hepatitis C, cirrhosis or hepatocellular carcinoma. The risk of vertical transmission is increased when mothers are co-infected with Human Immunodeficiency Virus (HIV) or possess a high viral load. There is no clear data supporting that mode of delivery increases or reduces risk. Breastfeeding is not associated with increased risk of transmission. Premature rupture of membranes, invasive procedures (such as amniocentesis), intrapartum events, or fetal scalp monitoring may increase risk of transmission. In pregnant patients, hepatitis C is diagnosed with a positive ELISA-3 and detectable Hepatitis C Virus (HCV) RNA viral load. Infants born to HCV-infected mothers should be tested for either HCV RNA on at least two separate occasions. Although prevention is not possible, there may be a role for newer direct acting anti-viral medications in the future.

## 1. Introduction

As of 2013, hepatitis C was the most common indication for liver transplant [1]. While incidence of hepatitis C has declined overall within the past 20 years due to multiple interventions such as screening of blood products, increased awareness, improved hygiene, and effective second-generation direct-acting antiviral therapy, there is concern that its prevalence may rise in the future with the worsening nationwide opioid epidemic within the United States [2,3]. In the United States, in particular, the incidence of the virus doubled between 2010 and 2014. This was partially attributed to an increase in injection drug use in rural areas [3]. Incidence may also increase due to lack of intervention for those who are acutely and chronically infected [3]. This poses the issue of increasing HCV infection in the pregnant population, which affects both the mother and her fetus during the pregnancy and can lead to chronic infection in the infant with downstream sequelae of cirrhosis and hepatocellular carcinoma. Currently, hepatitis C affects 8% of pregnant women globally [4]. With the development of new drug therapies that are highly effective and well-tolerated in non-pregnant individuals, there is also potential for these drugs to be used in pregnant patients with hepatitis C [5].

Due to potentially increasing incidence, aspects of hepatitis C in pregnancy were comprehensively reviewed to contribute to current research on the role of direct-acting anti-viral agents in pregnant patients with hepatitis C and the current controversy over the role of screening for hepatitis C in the pregnant population. To do so, the keywords “hepatitis C” and “hepatitis C in pregnancy” were searched for to obtain relevant articles published from 1995 to present within the PUBMED and Google Scholar search engines. In total, 67 articles were selected for reference. 

## 2. Epidemiology

The estimated global prevalence of hepatitis C is 1.5% to 2%, affecting up to 71 million people. Prevalence varies between high-income countries versus developing countries; in the latter, prevalence has been estimated to be up to 13% higher [6]. Hepatitis C is responsible for roughly 27% of cases of cirrhosis and 25% of cases of hepatocellular carcinoma [7]. In the pregnant population, the prevalence of serum HCV antibodies has been estimated in large cohorts as ranging between 0.1% and 3.6%. The prevalence of HCV RNA within the general population in the same cohorts has been estimated as between 42% and 72% [8]. The prevalence of hepatitis C in children acquired through vertical transmission is approximately 0.2–0.4% in the US and Europe and as high as 12–14% in certain regions in Africa [9,10]. In general, chronic hepatitis C is more frequent than acute hepatitis C in pregnant patients and affects up to 8% of pregnant women globally [3,4].

## 3. Clinical Course and Metabolic Features

The natural course of hepatitis C is a progression from acute hepatitis (defined as occurring in the first six months following exposure) to chronic hepatitis, which occurs in 55%–85% of patients. Those who do not progress to chronic illness spontaneously clear the infection [4,11]. Patients with acute and chronic hepatitis can be asymptomatic, although those with acute infection are more likely to present with jaundice, malaise, abdominal pain, anorexia, or nausea [4]. Up to 30% of patients with chronic hepatitis C who do not receive treatment progress to cirrhosis [4]. A study done by Rinaldi et al. describes that patients with HCV-related cirrhosis thereafter follow a course similar to cryptogenic cirrhosis in overweight/obese individuals, with the development of ascites, hepatocellular carcinoma, or death [11]. The risk of those with cirrhosis developing hepatocellular carcinoma is around 2–4% [11]. Median length of survival of these patients following diagnosis is less than two years [4]. 

Recent literature suggests that hepatitis C functions as a metabolic virus. Ress et al. explain that hepatitis C directly influences hepatic lipid metabolism, resulting in deposition of lipid droplets on hepatocytes [12]. Lipid droplets are targeted by the virus and influence its course [12]. Steatosis, found in 80% of patients with hepatitis C, signifies decreased beta oxidation, increased gluconeogenesis, and de novo lipogenesis [12]. Insulin resistance is both a direct result and an exacerbating factor for lipid droplet formation [12]. A review by Ballestri et al. supports the notion that hepatitis C functions as a metabolic virus by hypothesizing a correlation between hepatitis C and type 2 diabetes [13]. It mentions the similarities of hepatitis C-associated dysmetabolic syndrome (HCADS) with the metabolic syndrome associated with non-alcoholic fatty liver disease [13]. Lonardo et al. also mentions HCADS, identifying type 2 diabetes as an extra-hepatic manifestation of hepatitis C likely secondary to exacerbation of insulin resistance [14]. They suggest that the virus may also play a role in cardiac dysfunction and accelerated atherogenesis, which may play a role in maternal adverse outcomes associated with hepatitis C [14].

## 4. Differential Diagnosis

Acute hepatitis C can manifest with symptoms in up to 75% of infected patients [4]. In the pregnant population, these symptoms overlap with other liver abnormalities. The differential diagnosis of liver abnormalities in pregnancy fall within four major categories, including normal physiological liver changes during pregnancy, newly acquired liver disease prevalent in pregnancy, liver disease unique to pregnancy, and liver disease present prior to pregnancy [15]. Normally within pregnancy, circulatory changes result in a decreased percentage of cardiac output reaching the liver, which can result in impaired hepatic metabolism and clearing. Hormonal changes affect biliary smooth muscle contractility and transport, which are largely asymptomatic, but can result in cholestasis. Within this category of differential diagnoses, normal changes in liver chemistries include increased alkaline phosphatase and decreased albumin levels [15]. Newly acquired liver diseases in pregnancy include viral hepatitis and biliary disease such as cholelithiasis. Liver diseases observed only during pregnancy include intrahepatic cholestasis, preeclampsia, eclampsia, HELLP syndrome, and acute fatty liver of pregnancy. Liver abnormalities can also be seen in hyperemesis gravidarum in up to 50% of cases [15].

## 5. Modes of Transmission

Hepatitis C can be transmitted via transfusion of infected blood products (which is now rare in industrialized countries), sharing of needles among infected intravenous drug users, piercings and tattoos, intranasal drug use, sexual contact, unsterile medical injections and surgical procedures, and mother-to-child transmission (i.e., vertical transmission) [3,16,17]. Pregnant patients primarily acquire hepatitis C by either intravenous drug use or history of blood transfusions [8]. The virus can then be transmitted to the fetus of an infected mother by intrauterine, intrapartum, or postnatal routes [18,19,20].

Vertical transmission can occur during all three trimesters in mothers who are either infected only with hepatitis C or are co-infected with HIV. In studies conducted by Gibb et al. and Mok et al., results suggested that because postpartum transmission of the virus is rare, transmission likely occurs during the intrapartum period or in utero, respectively [21,22]. It typically occurs in patients with detectable viral loads, and occurs rarely if the maternal viral load is less than 1 × 10^5^ HCV RNA copies/mL plasma [18,19,20]. However, there are case reports of fetal transmission in mothers without detectable viremia [16]. There are inconsistent results from studies attempting to correlate viral load with risk of transmission [23].

Additional factors that have been studied to determine their influence on the risk of transmission between mother and fetus include co-infection with HIV, means of delivery, breast-feeding, and rupture of membranes [16,24]. Maternal co-infection with HIV has shown to strongly increase the risk of vertical transmission [6]. The risk of vertical transmission of the infection to children is greater than 5% for HIV-negative mothers whereas it is greater than 10% in HIV-positive mothers [6]. The impact of modes of delivery on risk of vertical transmission is inconsistent in the literature. While data supports an increased risk of transmission with prolonged rupture of membranes, other studies did not report an increased risk of transmission with vaginal delivery, which is associated with a more prolonged rupture of membranes than cesarean section. In the comparison of risk of vertical transmission associated with vaginal and emergent cesarean sections versus that with elective cesarean sections, studies have conflicting results. Some studies indicate an increased risk with the former (i.e., vaginal delivery and emergent cesarean sections) while another study indicates increased risk associated with the latter [23,25,26]. The Society for Maternal Fetal Medicine (SMFM) most recently refutes any association between mode of delivery and transmission of hepatitis C, as supported by the American College of Obstetricians & Gynecologists (ACOG) based upon a systematic review conducted by Cotrell et al. in 2013 [4,25]. Breastfeeding is not associated with increased risk of transmission unless done in the presence of cracked or bleeding nipples [26,27,28]. Premature rupture of membranes, invasive procedures (such as amniocentesis), intrapartum events, or fetal scalp monitoring that may lead to exposure of the infant to infected maternal blood may increase risk of transmission [16,29,30].

## 6. Patterns Observed in Hepatitis C-Infected Pregnant Patients

For those pregnant patients with chronic hepatitis C, alanine aminotransferase levels have been found to decrease and subsequently increase postpartum. This trend has partially been attributed to the release of endogenous interferon from the placenta, hemodilution, immune tolerance, or the influence of sexual hormones or immunosuppressive cytokines synthesized during pregnancy [8,31,32,33,34]. Many studies demonstrated a slight increase in average viral load as the infected mother progresses through her pregnancy, particularly in the second and third trimesters [33,35]. This increase is associated with the aforementioned decline in alanine aminotransferase. Some cases report a decline in the viral load of hepatitis C-infected mothers in the immediate postpartum phase and rarely, spontaneous resolution of the virus during the same phase [35,36]. Other studies report no significant change in viral load during and after pregnancy [34]. A prospective studying analyzing fibrosis scores in hepatitis C-infected mothers throughout pregnancy showed marginal increases in fibrosis with each passing trimester [37].

## 7. Neonatal and Maternal Outcomes Related to Chronic Hepatitis C

Some studies suggest that the fetuses of mothers infected with chronic hepatitis C are at increased risk for adverse neonatal outcomes. One study demonstrated that infants of hepatitis C-positive mothers were more likely to be small for gestational age, require assisted ventilation, be admitted to the intensive care unit, or have low birth weight [8,38]. Another study made similar inferences, suggesting that infants born to infected mothers are at risk for low birth weight, preterm birth, and congenital anomalies, although confounding factors, such as polysubstance abuse, were not controlled for [8,39]. A more recent study demonstrated impaired intrauterine fetal growth of infants born to hepatitis C-infected mothers [40].

Adverse maternal outcomes have also been identified, such as a higher risk of development of intrahepatic cholestasis [41,42,43]. Other adverse associations include gestational diabetes and preterm delivery [17,38,44]. These metabolic complications may be a result of the aforementioned role of hepatitis C as a metabolic virus [12,13,14]. A recent study conducted by Karampatou et al. suggests that premature ovarian senescence, characterized by a decline in anti-Müllerian hormone, is observed in women with hepatitis C [45]. The study also demonstrates higher rates of miscarriage in patients with hepatitis C as compared to those with hepatitis B [45].

Children who have acquired hepatitis C via vertical transmission can clear the infection, have persistent asymptomatic mild liver disease, or develop end-stage liver disease. The rate of spontaneous clearance of the infection is reportedly between 11% and 25% [16,46,47,48].

## 8. Issues Regarding Screening

Guidelines proposed by the ACOG, the Centers for Disease Control (CDC), the SMFM and the American College of Gastroenterology (ACG) recommend that pregnant women be screened for hepatitis C based on the presence of certain risk factors. These risk factors include: injection drug use, use of illicit intranasal drugs, treatment with long-term dialysis, experience with percutaneous or parenteral exposure in unregulated settings (such as tattoos received outside of licensed parlors or unhygienic medical procedures), receipt of blood transfusions or organ transplants before 1992, receipt of certain blood products prior to 1987, receipt of blood products from an HCV-positive donor, history of incarceration, risk of HIV, and history of chronic liver disease of unknown etiology [4,49,50]. The same applies to the general population, including women of child-bearing age [49,50]. Although no guidelines have been proposed by the Asian Pacific Association for the Study of the Liver regarding screening, it is recommended based on geographical location and risk [51]. Similarly, the European Association for the Study of the Liver (EASL) recommends that screening be tailored to each region or nation based on local epidemiology and prevalence of risk [52].

Controversy has arisen with the advent of direct-acting antiviral therapy [4,53]. With the potential for such agents to cure hepatitis C in non-pregnant patients and possibly pregnant patients, there have been proponents for universal screening for hepatitis C in pregnancy. However, hepatitis C screening has not proven to be cost effective and is not generally recommended [54,55]. In addition, there may be no utility in screening as no prevention (e.g., vaccination) or definite treatment is available for women infected with hepatitis C during pregnancy [35,55,56]. One large study conducted in London by Selvapatt, et al. refuted these findings, suggesting that screening and treatment is feasible with acceptable cost [57]. This study, however, has not been replicated elsewhere.

Guidelines proposed by the American College of Pediatrics and the CDC recommend screening infants born to HCV-infected mothers either for HCV RNA on at least two separate occasions when the infant is older than 1 month of age; or to screen for anti-HCV antibodies when the infant is older than 18 months [4,58].

## 9. Diagnosis

For pregnant patients who test positive for HCV antibodies via ELISA-3, HCV RNA should be obtained. If a viral load is present, confirming active infection, the viral genotype can also be determined [10,54]. The absence of HCV RNA may indicate either a cleared infection or a false positive. In order to distinguish between the two, the recombinant immunoblot assay (i.e., RIBA) can be performed and utilized in research, but is no longer used clinically [10].

For infants born to HCV-positive mothers, the CDC recommendations are as aforementioned [10,18]. Screening is completed after the first year of life due to the presence of maternal antibodies that passively move across the placenta from mother to fetus, which may be falsely mistaken for the infant’s [10]. If testing must be done or an infant is found to be positive for hepatitis C antibodies, HCV RNA should be ordered to confirm or refute the diagnosis [59]. 

## 10. Prevention and Treatment

Hepatitis C was previously treated with a combination of pegylated interferon alpha and ribavirin due to decreased sustained virologic response with either agent alone. It has not been used in the pregnant population due to established effects on fetal growth and teratogenicity, respectively [60,61,62]. Ribavirin was found to cause abnormalities of the skull, palate, eye, jaw, limbs, skeleton, and gastrointestinal tract in animal models. Interferon was found to have abortifacient properties in animal studies, with a risk of causing infertility [62]. This combination is also contraindicated in the postpartum period due to its transmission through breast milk and psychiatric side effects associated with pegylated interferon alpha that may hinder the mother’s ability to appropriately care for her child [35,62]. 

There are no vaccinations or treatments available for prevention. Studies do not necessarily support cesarean section as a means of preventing vertical transmission [24,63]. Newer direct acting antiviral agents (DAAs) such as sofosbuvir, ledipasvir, daclatasvir, simeprevir, paritaprevir, ombitasvir, and dasabuvir have the potential to prevent vertical transmission by quickly suppressing HCV RNA levels, which, in high levels, have demonstrated an increased risk of vertical transmission. These agents have also shown sustained virologic response with minimal toxicity [62,63]. Data extracted from animal studies done with these agents have showed minimal teratogenic effects, allowing for some of these agents to be labeled as category B by the Food & Drug Administration (FDA) [64]. Ledipasvir and sofosbuvir, in particular, demonstrated no fetal harm in animal studies, resulting in their approval as Category B by the [5]. In comparison, simeprevir is classified as category C, as animal studies showed reduced fetal weights, in utero fetal losses, early maternal deaths with high exposure, and fetal skeletal variations [5]. Still, combinations of ledipasvir and sofosbuvir, or paritprevir, ombitasvir, and dasabuvir are exhibiting superior results in phase III trials as compared to prior therapies [5]. However, due to limited data as to use in pregnancy in humans and the presence of teratogenic effects, although minimal, in animal studies, there is no particular consensus as to guidelines for treatment [62,63,64,65]. The EASL recommends individual DAAs or combinations thereof [52]. The American Association for the Study of Liver Diseases, in partnership with the Infectious Diseases Society of America, have created separate treatment guidelines utilizing DAAs, which are also supported by the CDC [66,67]. ACG does not condone treatment of hepatitis C in the pregnant patient [50].

## 11. Conclusions

Hepatitis C in pregnancy may increase with the current nationwide opioid epidemic [2,3]. It is crucial to understand hepatitis C in pregnancy due to the potential for increased prevalence, vertical transmission, risk of adverse maternal and neonatal outcomes, and subsequent sequelae such as chronic hepatitis C, cirrhosis, and hepatocellular carcinoma in the infant. The availability of new, potentially curative therapies will also likely shape the approach to treating peripartum HCV infection in both pregnant women and infants in the coming years, although additional prospective studies will be required. 

## References

[1] Martin P., DiMartini A., Feng S., Brown R., Fallon M. (2013). Evaluation for liver transplantation in adults: 2013 practice guideline by the AASLD and the American Society of Transplantation. Hepatology.

[2] Klevens R.M., Hu D.J., Jiles R., Holmberg S.D. (2012). Evolving epidemiology of hepatitis C virus in the United States. Clin. Infect. Dis..

[3] World Health Organization (2014). Guidelines for the Screening, Care and Treatment of Persons with Hepatitis C Infection. http://apps.who.int.revproxy.brown.edu/iris/bitstream/10665/111747/1/9789241548755_eng.pdf?ua=1.

[4] Hughes B.L., Page C.M., Kuller J.A. (2017). Hepatitis C in pregnancy: Screening, treatment, and management. Am. J. Obstet. Gynecol..

[5] Kanninen T.T., Dieterich D., Asciutti S. (2015). HCV vertical transmission in pregnancy: New horizons in the era of DAAs. Hepatology.

[6] Benova L., Mohamoud Y.A., Calvert C., Abu-Raddad L.J. (2014). Vertical Transmission of Hepatitis C Virus: Systematic Review and Meta-analysis. Clin. Infect. Dis..

[7] Alter M.J. (2007). Epidemiology of hepatitis C virus infection. World J. Gastroenterol..

[8] Floreani A. (2013). Hepatitis C and pregnancy. World J. Gastroenterol..

[9] Pawlowska M. (2015). Pegylated IFN-α-2a and ribavirin in the treatment of hepatitis C infection in children. Expert Opin. Drug Saf..

[10] Muñoz-Gámez J.A., Salmerón J., Ruiz-Extremera Á. (2016). Hepatitis C during pregnancy, vertical transmission and new treatment possibilities. Med. Clín. (Engl. Ed.).

[11] Rinaldi L., Nascimbeni F., Giordano M., Masetti C., Guerrera B., Amelia A., Fascione M.C., Ballestri S., Romagnoli D., Zampino R. (2017). Clinical features and natural history of cryptogenic cirrhosis compared to hepatitis C virus-related cirrhosis. World J. Gastroenterol..

[12] Ress C., Kaser S. (2016). Mechanisms of intrahepatic triglyceride accumulation. World J. Gastroenterol..

[13] Ballestri S., Nascimbeni F., Romagnoli D., Baldelli E., Targher G., Lonardo A. (2016). Type 2 Diabetes in non-alcoholic fatty liver disease and hepatitis C virus infection—Liver: The “Musketeer” in the spotlight. Int. J. Mol. Sci..

[14] Lonardo A., Ballestri S., Guaraldi G., Nascimbeni F., Romagnoli D., Zona S., Targher G. (2016). Fatty liver is associated with an increased risk of diabetes and cardiovascular disease—Evidence from three different disease models: NAFLD, HCV and HIV. World J. Gastroenterol..

[15] Kia L., Rinella M.E. (2013). Interpretation and management of hepatic abnormalities in pregnancy. Clin. Gastroenterol. Hepatol..

[16] Tovo P.-A., Calitri C., Scolfaro C., Gabiano C., Garazzino S. (2016). Vertically acquired hepatitis C virus infection: Correlates of transmission and disease progression. World J. Gastroenterol..

[17] Rac M.W., Sheffield J.S. (2014). Prevention and management of viral hepatitis in pregnancy. Obstet. Gynecol. Clin..

[18] Le Campion A., Larouche A., Fauteux-Daniel S., Soudeyns H. (2012). Pathogenesis of hepatitis C during pregnancy and childhood. Viruses.

[19] Indolfi G., Resti M. (2009). Perinatal transmission of hepatitis C virus infection. J. Med. Virol..

[20] Elrazek A., Amer M., El-Hawary B., Salah A., Bhagavathula A.S., Alboraie M., Saab S. (2017). Prediction of HCV vertical transmission: What factors should be optimized using data mining computational analysis. Liver Int..

[21] Gibb D.M., Goodall R.L., Dunn D.T., Healy M., Neave P., Cafferkey M., Butler K. (2000). Mother-to-child transmission of hepatitis C virus: Evidence for preventable peripartum transmission. Lancet.

[22] Mok J., Pembrey L., Tovo P.A., Newell M.L. (2005). When does mother to child transmission of hepatitis C virus occur?. Arch. Dis. Child.-Fetal Neonatal Ed..

[23] Mast E.E., Hwang L.Y., Seto D.S., Nolte F.S., Nainan O.V., Wurtzel H., Alter M.J. (2005). Risk factors for perinatal transmission of hepatitis C virus (HCV) and the natural history of HCV infection acquired in infancy. J. Infect. Dis..

[24] Yeung C.-Y., Lee H.-C., Chan W.-T., Jiang C.-B., Chang S.-W., Chuang C.-K. (2014). Vertical transmission of hepatitis C virus: Current knowledge and perspectives. World J. Hepatol..

[25] Cottrell E.B., Chou R., Wasson N., Rahman B., Guise J.M. (2013). Reducing risk for mother-to-infant transmission of hepatitis C virus: A systematic review for the US Preventive Services Task Force. Ann. Intern. Med..

[26] European Paediatric Hepatitis C Virus Network (2005). A significant sex—But not elective cesarean section—effect on mother-to-child transmission of hepatitis C virus infection. J. Infect. Dis..

[27] Moyer V.A. (2013). Screening for hepatitis C virus infection in adults: US Preventive Services Task Force recommendation statement. Ann. Intern. Med..

[28] Chou R., Cottrell E.B., Wasson N., Rahman B., Guise J.-M. (2012). Screening for Hepatitis C Virus Infection in Adults [Internet].

[29] Shaheen M.A., Idrees M. (2015). Evidence-based consensus on the diagnosis, prevention and management of hepatitis C virus disease. World J. Hepatol..

[30] Post J.J. (2017). Update on hepatitis C and implications for pregnancy. Obstet. Med..

[31] Ohto H., Terazawa S., Sasaki N., Sasaki N., Hino K., Ishiwata C., Kako M., Ujiie N., Endo C., Matsui A. (1994). Transmission of hepatitis C virus from mothers to infants. N. Engl. J. Med..

[32] Wejstål R., Widell A., Norkrans G. (1998). HCV-RNA levels increase during pregnancy in women with chronic hepatitis C. Scand. J. Infect. Dis..

[33] Gervais A., Bacq Y., Bernuau J., Martinot M., Auperin A., Boyer N., Kilani A., Erlinger S., Valla D., Marcellin P. (2000). Decrease in serum ALT and increase in serum HCV RNA during pregnancy in women with chronic hepatitis C. J. Hepatol..

[34] Paternoster D.M., Santarossa C., Grella P., Palu G., Baldo V., Boccagni P., Floreani A. (2001). Viral load in HCV RNA—positive pregnant women. Am. J. Gastroenterol..

[35] Prasad M.R., Honegger J.R. (2013). Hepatitis C virus in pregnancy. Am. J. Perinatol..

[36] Watanabe H., Saito T., Shinzawa H., Okumoto K., Hattori E., Adachi T., Takeda T., Sugahara K., Ito J., Saito K. (2003). Spontaneous elimination of serum hepatitis C virus (HCV) RNA in chronic HCV carriers: A population-based cohort study. J. Med. Virol..

[37] Money D., Boucoiran I., Wagner E., Dobson S., Kennedy A., Lohn Z., Krajden M., Yoshida E.M. (2014). Obstetrical and neonatal outcomes among women infected with hepatitis C and their infants. J. Obstet. Gynaecol. Can..

[38] Pergam S.A., Wang C.C., Gardella C.M., Sandison T.G., Phipps W.T., Hawes S.E. (2008). Pregnancy complications associated with hepatitis C: Data from a 2003–2005 Washington state birth cohort. Am. J. Obstet. Gynecol..

[39] Connell L.E., Salihu H.M., Salemi J.L., August E.M., Weldeselasse H., Mbah A.K. (2011). Maternal hepatitis B and hepatitis C carrier status and perinatal outcomes. Liver Int..

[40] Huang Q.T., Hang L.L., Zhong M., Gao Y.F., Luo M.L., Yu Y.H. (2016). Maternal HCV infection is associated with intrauterine fetal growth disturbance: A meta-analysis of observational studies. Medicine.

[41] Geenes V., Williamson C. (2009). Intrahepatic cholestasis of pregnancy. World J. Gastroenterol..

[42] Paternoster D.M., Fabris F., Palù G., Santarossa C., Bracciante R., Snijders D., Floreani A. (2002). Intra-hepatic cholestasis of pregnancy in hepatitis C virus infection. Acta Obstet. Gynecol. Scand..

[43] Locatelli A., Roncaglia N., Arreghini A., Bellini P., Vergani P., Ghidini A. (1999). Hepatitis C virus infection is associated with a higher incidence of cholestasis of pregnancy. BJOG Int. J. Obstet. Gynaecol..

[44] Reddick K.L.B., Jhaveri R., Gandhi M., James A.H., Swamy G.K. (2011). Pregnancy outcomes associated with viral hepatitis. J. Viral Hepat..

[45] Karampatou A., Han X., Kondili L.A., Taliani G., Ciancio A., Morisco F., Critelli R.M., Baraldi E., Bernabucci V., Troshina G. (2018). Premature ovarian senescence and a high miscarriage rate impair fertility in women with HCV. J. Hepatol..

[46] Bortolotti F., Verucchi G., Cammà C., Cabibbo G., Zancan L., Indolfi G., Giacchino R., Marcellini M., Marazzi M.G., Barbera C. (2008). Long-term course of chronic hepatitis C in children: From viral clearance to end-stage liver disease. Gastroenterology.

[47] European Paediatric Hepatitis C Virus Network (2005). Three broad modalities in the natural history of vertically acquired hepatitis C virus infection. Clin. Infect. Dis..

[48] Yeung L.T.F., To T., King S.M., Roberts E.A. (2007). Spontaneous clearance of childhood hepatitis C virus infection. J. Viral Hepat..

[49] Centers for Disease Control and Prevention (2011). Sexually transmitted diseases treatment guidelines, 2010. Ann. Emerg. Med..

[50] Tran T.T., Ahn J., Reau N.S. (2016). ACG clinical guideline: Liver disease and pregnancy. Am. J. Gastroenterol..

[51] Omata M., Kanda T., Wei L., Yu M.L., Chuang W.L., Ibrahim A., Lesmana C.R.A., Sollano J., Kumar M., Jindal A. (2016). APASL consensus statements and recommendations for hepatitis C prevention, epidemiology, and laboratory testing. Hepatol. Int..

[52] European Association for the Study of the Liver (2018). EASL Recommendations on Treatment of Hepatitis C 2018. J. Hepatol..

[53] Orkin C., Jeffery-Smith A., Foster G.R., Tong C.W. (2016). Retrospective hepatitis C seroprevalence screening in the antenatal setting—Should we be screening antenatal women?. BMJ Open.

[54] Plunkett B.A., Grobman W.A. (2005). Routine hepatitis C virus screening in pregnancy: A cost-effectiveness analysis. Am. J. Obstet. Gynecol..

[55] Page C.M., Hughes B.L., Rhee E.H., Kuller J.A. (2017). Hepatitis C in Pregnancy: Review of Current Knowledge and Updated Recommendations for Management. Obstet. Gynecol. Surv..

[56] Mitchell A.E., Colvin H.M. (2010). Hepatitis and Liver Cancer: A National Strategy for Prevention and Control of Hepatitis B and C.

[57] Selvapatt N., Ward T., Bailey H., Bennett H., Thorne C., See L.M., Tudor-Williams G., Thursz M., McEwan P., Brown A. (2015). Is antenatal screening for hepatitis C virus cost-effective? A decade’s experience at a London centre. J. Hepatol..

[58] Polywka S., Pembrey L., Tovo P.A., Newell M.L. (2006). Accuracy of HCV-RNA PCR tests for diagnosis or exclusion of vertically acquired HCV infection. J. Med. Virol..

[59] Resti M., Bortolotti F., Vajro P., Maggiore G. (2003). Guidelines for the screening and follow-up of infants born to anti-HCV positive mothers. Dig. Liver Dis..

[60] Chutaputti A. (2000). Adverse effects and other safety aspects of the hepatitis C antivirals. J. Gastroenterol. Hepatol..

[61] Valladares G., Chacaltana A., Sjogren M.H. (2010). The management of HCV-infected pregnant women. Ann. Hepatol..

[62] Spera A.M., Eldin T.K., Tosone G., Orlando R. (2016). Antiviral therapy for hepatitis C: Has anything changed for pregnant/lactating women?. World J. Hepatol..

[63] Chehreh M.E.G., Tabatabaei S.V., Khazanehdari S., Alavian S.M. (2011). Effect of cesarean section on the risk of perinatal transmission of hepatitis C virus from HCV-RNA+/HIV− mothers: A meta-analysis. Arch. Gynecol. Obstet..

[64] Bernstein H.B., Dunkelberg J.C., Leslie K.K. (2018). Hepatitis C in Pregnancy in the Era of Direct-acting Antiviral Treatment: Potential Benefits of Universal Screening and Antepartum Therapy. Clin. Obstet. Gynecol..

[65] Barritt A.S., Jhaveri R. (2018). Treatment of Hepatitis C during Pregnancy-Weighing the Risks and Benefits in Contrast to HIV. Curr. HIV/AIDS Rep..

[66] Centers for Disease Control and Prevention (2018). Hepatitis C FAQs for Health Professionals.

[67] American Association for the Study of Liver Diseases, & Infectious Diseases Society of America (2018). Recommendations for Testing, Managing, and Treating Hepatitis C. http://www.hcvguidelines.org.

